# Effect of Clonal Selection on *Daphnia* Tolerance to Dark Experimental Conditions

**DOI:** 10.1371/journal.pone.0159628

**Published:** 2016-07-19

**Authors:** Sandra J. Connelly, James A. Stoeckel, Robert A. Gitzen, Craig E. Williamson, Maria J. González

**Affiliations:** 1 Thomas H. Gosnell School of Life Sciences, Rochester Institute of Technology, Rochester, NY 14623, United States of America; 2 School of Fisheries, Aquaculture and Aquatic Sciences, College of Agriculture, Auburn University, Auburn, AL 36849, United States of America; 3 Department of Biology, Miami University, Oxford, OH 45056, United States of America; 4 School of Forestry and Wildlife Sciences, Auburn University, Auburn, AL 36849, United States of America; Federal University of Rio de Janeiro, BRAZIL

## Abstract

Recent studies have demonstrated substantial effects of environmental stress that vary among clones. Exposure to ultraviolet radiation (UV) is an important abiotic stressor that is highly variable in aquatic ecosystems due to diel and seasonal variations in incident sunlight as well as to differences in the UV transparency of water among water bodies, the depth distribution of organisms, and the ability of organisms to detect and respond to UV. In contrast to the convention that all UV is damaging, evidence is accumulating for the beneficial effects of exposure to low levels of UV radiation. Whereas UV has been frequently observed as the primary light-related stressor, herein we present evidence that dark conditions may be similarly “stressful” (reduction of overall fitness), and stress responses vary among clones of the freshwater crustacean *Daphnia parvula*. We have identified a significant relationship between survivorship and reduced fecundity of clones maintained in dark conditions, but no correlation between tolerance of the clones to dark and UV radiation. Low tolerance to dark conditions can have negative effects not only on accumulated stresses in organisms (e.g. the repair of UV-induced damage in organisms with photolyase), but potentially on the overall physiology and fitness of organisms. Our results support recent evidence of the beneficial effects of low-level UV exposure for some organisms.

## Introduction

Natural and anthropogenic changes in abiotic factors and the stresses that they induce in organisms have long been a focus of researchers. Solar ultraviolet radiation (UVR) exposure of these systems and their inhabitants is a well-known stressor (e.g. [1]). Decreased stratospheric ozone historically has been reported as altering UVR exposures at the Earth’s surface. While isolated studies have suggested a stabilization of the total ozone column (TOC) in recent years, the variability in the TOC globally due to considerable atmospheric and climatic changes and shortcomings of the measurements themselves should not be overlooked [2].

DNA is thought to be the primary target of UVR damage and induction of damage in DNA is linearly related to UVR exposure (dose) in isolated DNA [3], but has been more associated with overall physiological effects in multiple organisms, particularly in high UVR regions (e.g. [4]). Further, recent studies have suggested sublethal effects of UVR exposure could be the most critical to the stability and evolution of populations, particularly aquatic species in which diel migration patterns are altered [5–6], in which the need for photoprotective mechanisms are taxed with changing UVR (e.g. [7]), and that experience increased physiological stressors (e.g. respiration stress [8]).

Conversely, recent studies suggest that a wide range of organisms may benefit from exposure to low levels of UV radiation [9]. The reasons for this are likely multifaceted. However, indications that increased UVR may act to decrease pathogens in ecosystems concomitantly may improve the overall health of some organisms [10]. Given the evidence for positive effects of UVR and the fundamental role of light (e.g. driving photosynthesis, necessity for vision, regulating predator-prey interactions, production of vitamin D, etc.), the potential advantages of light availability must be further considered. Whereas most studies of UVR and light effects on organisms in both laboratory and field studies include a “dark” control (typically either complete darkness or low-level light), the possibility that the absence of light in and of itself may be detrimental to the organisms requires serious consideration. Here we investigate the potential detrimental effects of total darkness where no UVR or other light is present. Specifically, we address the following:

1. Is there evidence for detrimental effects of total darkness in the common crustacean grazer *Daphnia parvula*?
2. Does low tolerance of dark conditions vary among clones of *D*. *parvula*?
3. Are survival rates of *D*. *parvula* exposed to long periods of dark conditions related to reproductive output or UV tolerance?

## Materials and Methods

To investigate the effects of dark conditions on the survival and reproduction of *Daphnia parvula* clones, we conducted two primary experiments: a dark tolerance experiment and a UV tolerance experiment. We conducted a preliminary experiment to identify “dark tolerant” and “dark intolerant” clone lines for use in the primary experiments.

### Daphnia

*Daphnia* were acquired initially from natural populations and were cultured as laboratory clonal population lines for more than six months prior to the start of the experiments presented herein. *Daphnia parvula* were collected from Acton Lake, in Hueston Woods State Park (39°34'21.50”N, 84°45'03.76”W), College Corner, OH. No specific permissions were required for these collections as Acton Lake is a public fishing and boating lake in Southwestern Ohio, and no samples of endangered or protected species were collected in connection to this study. Ten clones of *D*. *parvula* were cultured under a 16:8 light:dark (16:8 L:D) regime at 20°C (Precision Scientific 818 Illuminated Incubator, Chicago, IL, USA; PAR = 54 μEin/m^2^s). All cultures were maintained in synthetic freshwater (pH 7.0; US EPA 2002) and fed *Selenastrum* at 6x10^5^ cells/mL culture every 72 h. *D*. *parvula* were maintained as clonal lines established from individual resting eggs from different ephippia as part of a separate study [11].

### Experimental chamber

All dark tolerance experiments were conducted in the same controlled environmental chamber (Environmental Room, Environmental Growth Chambers, Chagrin Falls, OH). Beakers were held on four shelves (two shelves per treatment), where each shelf was wrapped in black felt fabric to exclude ambient light from adjacent treatments. The diel shelves were equipped with a bank of 2 CoolWhite bulbs (Philips, 32W, unfiltered) on a 14:10 L:D cycle. The “dark” shelves were not equipped with a light and light levels were < 1 μEin/m^2^s as quantified using a spherical detector QSL-2101 (Biospherical Instruments, San Diego, CA, USA). For the UV-tolerance experiment the UVR exposure of the clone lines was tested in a separate environmentally controlled chamber (description below), however, the dark controls were maintained in the same Environmental Room just described.

### Preliminary experiment

To determine if there was clonal variability of the *Daphnia parvula* to dark conditions, we conducted a dark control treatment experiment with three clones of *D*. *parvula* (A31, A60, A69). Six 50 ml beakers were filled with 30 mL culture media (1 L of culture media = *Selenastrum* at 10^5^ cells L^-1^, 1 mL Yeast-Trout Chow [YTC] *Daphnia* Feed Mixture [Aquatic BioSystems, Fort Collins, CO, USA], and EPA Synthetic Freshwater, pH 7.0 [12]). Thirty individuals of a given clone were then randomly assigned to the beakers (5 *Daphnia* per beaker). Each beaker was then randomly assigned to one of two treatments: dark or diel (16:8 L:D; light source = Philips Premium Cool White® 40W fluorescent bulbs not filtered), in an Environmental Room (Environmental Growth Chambers, Chagrin Falls, OH). With a total of three beakers per clone per treatment, adult survival was assessed, and the survivors were transferred to a new 50 ml beaker (filled as above) every 24 hrs for 5 days, whereby fully replenishing food and nutrients daily. Any neonates were discarded with the beaker change. We used a two-sample T-test (separate variance) to test for differences in survival between treatments for each individual clone.

### Dark tolerance experiment

To extend the preliminary experiment investigating the clonal variability of three *D*. *parvula* clones to dark conditions, 10 newly hatched neonates were collected from each of two females from 10 clonal lines within the same 24 hr period. Neonates were maintained on a 14:10 L:D cycle at 20°C and provided with fresh culture water, daily, containing 10^5^ cells L^-1^ *Selenastrum sp*. Neonates were reared for 10 days, at which time most had become gravid. Each female was held individually in a 50 ml beaker containing 30 mL culture media (1 L of culture media = *Selenastrum* at 10^5^ cells L^-1^, 1 mL Yeast-Trout Chow [YTC] *Daphnia* Feed Mixture [Aquatic BioSystems, Fort Collins, CO, USA], and EPA Synthetic Freshwater, pH 7.0 [12]). The females were assessed for survival and transferred to a new 50 ml beaker (filled as previously described) every 24 hrs for the duration of the experiment. At day 10, each female was randomly assigned to one of two treatments: a diel (14:10 L:D) and “dark” (no light except during brief sampling checks).

Every 24 hrs for seven days, each beaker was examined for adult survival (presence/absence of a visible heartbeat) and presence of neonates. Adults were transferred to a clean beaker containing culture media and food, and neonates were counted and discarded. Beakers were held in shallow trays (20 beakers/tray) and each tray was randomly assigned to a new position on the appropriate treatment shelves after each daily observation.

We used a Bayesian generalized linear mixed-effects model (GLMM) with a binomial error distribution to analyze the number of *Daphnia* still alive on Day 7 out of the number of *Daphnia* observed for each treatment x clone combination. This Bayesian approach facilitated straightforward quantification of variation between clones within and across treatments. Logit (probability of survival to Day 7) was modeled as a linear function of an intercept (β_0_, which estimated expected survival in the light treatment), a fixed effect of dark treatment consistent across clones (β_Dark_), a random effect of clone consistent across treatments (b_clone j_), and an observation-level (residual) random effect (b_clone j * treatment i_) to account for among-clone variation in treatment effects as well as other unexplained over dispersion. Random effects of clone were assumed to follow a Normal (mean = 0, variance = σ^2^_clone_) distribution, while the residual random effects were modeled with Normal (0, σ^2^_residual/treatment_) distribution such that the residual logit-scale variation was allowed to be heterogeneous between the two treatments (σ^2^_residual/treatment = light_ vs. σ^2^_residual/treatment = dark_). Prior distributions for the square root of the random effects variance parameters were specified to be Uniform (0,10) distributions, and prior distributions for β_0_ and β_Dark_ were specified as normal distributions with mean = 0, variance = 100.

Our Bayesian analysis was conducted in WinBUGS 1.4.3 [13] through R 3.10 (R Core Team 2014) using package R2WinBUGS [14]. Parameter posterior distributions were estimated from 3 Markov chain Monte Carlo (MCMC) chains with 95,000 samples retained per chain after a 50,000-sample burn-in per chain. We assessed MCMC convergence graphically and with the Gelman-Rubin statistic; convergence and mixing of chains was satisfactory (e.g. Gelman-Rubin statistic < 1.01 for all parameters). Based on back-transformation of relevant fixed and random terms in each MCMC iteration we calculated posterior distributions for population-wide average expected 7-day survival by treatment (light only vs. dark) and for clone-specific estimated survival by treatment. Posterior distributions of parameters were summarized with the mean and (0.025, 0.0975) percentiles (i.e. 95% credible intervals, Cr.I.) of the MCMC samples for each parameter.

We calculated the fecundity of each individual surviving until the end of the experiment by summing the number of neonates produced by that individual through day 7. We then used a two-sample T-test to test for differences in neonate production between treatments within each clonal line. To examine the relationship between dark treatment survival and change in fecundity under dark conditions, we regressed survivorship of each clone at Day 7 against L:D treatment / D treatment fecundity.

### UV tolerance experiment

To determine if there was a correlation between the tolerance of *Daphnia parvula* to dark conditions and their ability to survive UV-B, 6 clone lines exhibiting a range in apparent dark tolerance (see Results) were exposed to UV-B under controlled laboratory conditions. The lines were selected based on the results of the dark tolerance experiment and included lines that were qualified as having a low, intermediate, and high tolerance for the dark conditions. Each clonal population was run in a UV-B experiment three times over the course of a two-week period (total of 18 UV-B experiments).

**Fig 1:**
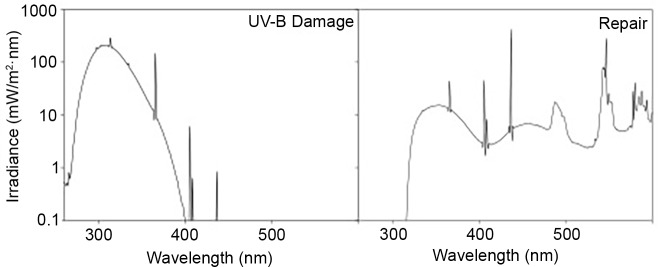
Emission spectra. The emission spectra for the lamp system used to induce DNA damage (left, UV-B Damage) and to photoreactivate damage after induction (right, Repair). This lamp design has been used in many laboratory-based UV studies of freshwater species and has been found to be highly applicable and comparable to what is observed in natural systems (e.g. [15] and its references).

UV-B and photoreactivating radiation (PRR) exposures were conducted in a phototron housed within an environmentally controlled chamber (Environmental Room, Environmental Growth Chambers, Chagrin Falls, OH) at 20°C. For a complete description of the UVR-lamp system see Williamson et al, 2001. The UV-B source consisted of three Spectronics Spectroline BLE-1T158 15W lamps (Westbury, NY, USA) yielding a spectral output of 281–405 nm (for damage radiation emission spectrum see Fig 1, UV-B Damage). Quartz Petri dishes (Quartz Scientific, Fairport Harbor, OH, USA) containing 5 *Daphnia* and 30 mL synthetic freshwater were placed inside black collars on a rotating platform (2 rpm) beneath the UV-B lamps to better control the UVR irradiance field and to assure uniform exposures. Samples were exposed for 12 hrs shielded with one of three different neutral density screens (stainless wire mesh, McMaster Carr, Princeton, NJ, USA) transmitting 28, 48, or 62% and yielding a total dose of 18, 32, and 41 kJ m^-2^, respectively. The doses used are equivalent to less than a full day of solar radiation at the water surface during summer solstice and average ozone conditions at 41° N latitude (full day exposure = 55 kJ m^-2^; [15]) and selected based on previous work with a mixed population of *D*. *parvula* that showed overall lower tolerance to UV-B. All UV-B exposures were conducted for the same length of time and in the same culture conditions with total UV-B dose as the only variable.

As some of the *D*. *parvula* used in this experiment were found to be dark intolerant in the experiment previously described, the experimental design was set up to deliver the PRR necessary for survival. The *Daphnia* were exposed to PRR from below while exposed to the UV-B from above [16]. The PRR consisted of two Q-Panel UV-A 340 lamps (Q-Panel Labs, Cleveland, OH, USA) and two CoolWhite lamps (Sylvania Premium CoolWhite 40W) filtered through two sheets of 12-μm thickness Mylar®D (E.I. Dupont & Co., Inc, Wilmington, DE, USA) (repair radiation emission spectrum, Fig 1).

Following exposure, all animals were maintained on a 14:10 L:D cycle at 20°C for 5 days and fed *Selenastrum* (0.25 mL = 2.5x10^4^ cells) every 24 h post exposure. Survival was recorded at each feeding by the presence/absence of a visual heartbeat. Control animals (no UV-B irradiation exposure) of clone were maintained under 14:10 L:D cycle at 20°C conditions for the duration of the experiment and their survival exceeded 90% for the duration of the experiment.

We used ANOVA followed by Tukey (SYSTAT 12, Systat Software, Inc. San Jose, CA) to test for differences in survival between clones at Day 5 post exposure for each of the three UV levels tested. Linear regression (SigmaPlot 10.0, Systat Software, Inc. San Jose, CA) was used to test for correlations between proportion survival of UV exposure and proportion survival of dark treatment.

## Results

### Preliminary dark tolerance experiment (3 clones)

**Fig 2:**
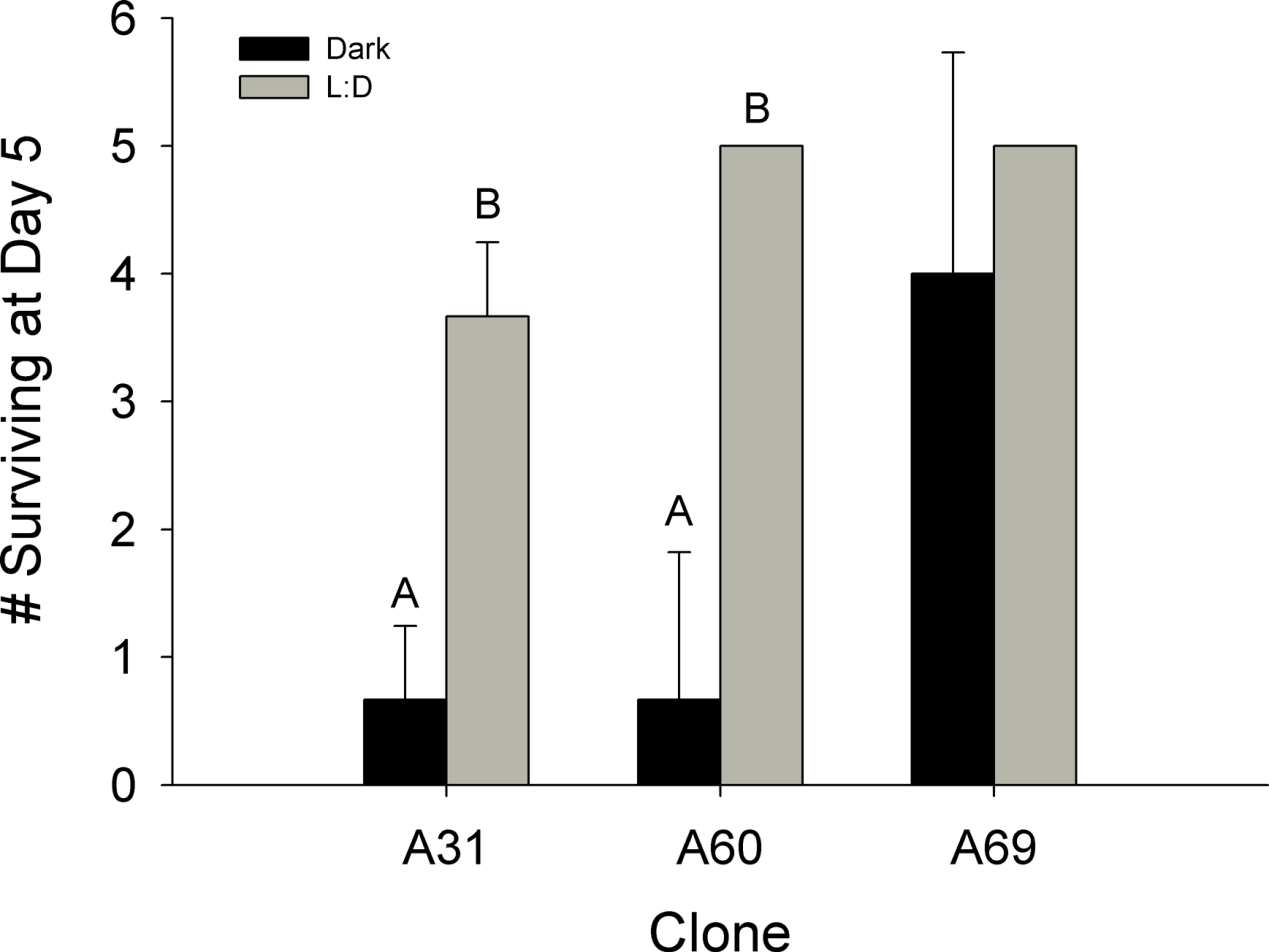
Survival of *D*. *parvula*. Number *Daphnia parvula* surviving to Day 5 for each of three clones held under dark and L:D conditions. Capital letters designate significant statistical differences between treatments within a clone. Effects of dark conditions on only some clones should be noted.

There was a significant difference between the mean number of surviving *Daphnia parvula* at day 5 in the dark (0.667 ± 0.577) compared to diel (3.667 ± 0.577) treatment for clone A31; t(4) = -6.364, *P* = 0.003 (Fig 2). Similarly, fewer *D*. *parvula* survived to day 5 in the dark (0.667 ± 1.155) as compared to diel (4.963 ± 0.055) treatment for clone A60; t(2.009) = -6.438, *P* = 0.023. However survival did not vary for clone A69 between dark (4.000 ± 1.732) and diel (5.00 ± 0) treatments; t(2) = -0.999, *P* = 0.423 (Fig 2).

### Dark tolerance experiment (10 clones)

**Fig 3:**
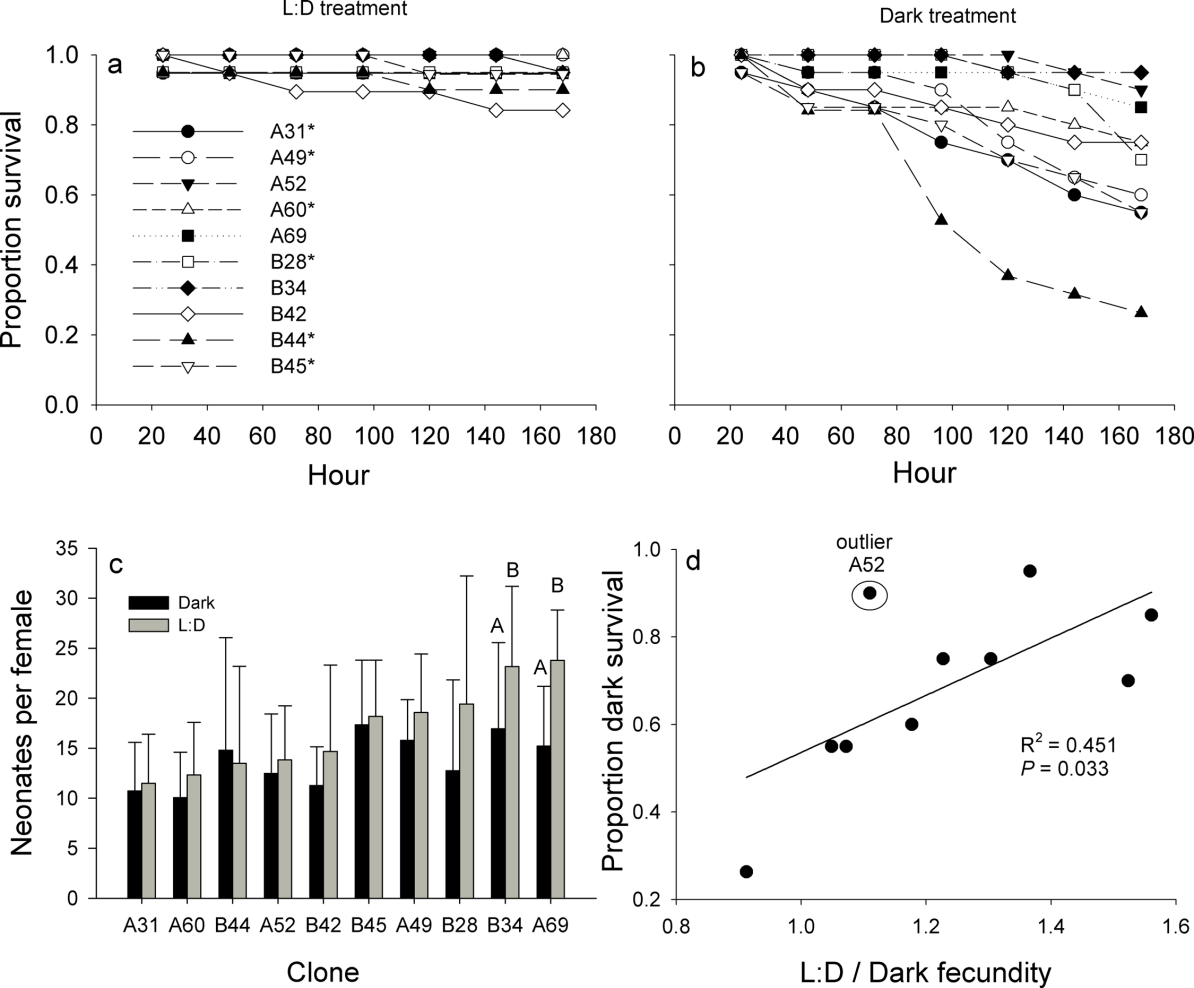
Dark tolerance fitness. Survival through time for ten clones in the a) L:D treatment and b) dark treatment. c) Mean cumulative number of neonates produced by surviving *Daphnia* in the dark and diel treatments. Capital letters indicate statistically significant differences between treatments for a given clone. d) Relationship between proportion survival in the dark treatment and proportional difference in fecundity between treatments (L:D / dark fecundity). Each data point represents a clone. A value of 1 on the Y-axis represents equal fecundity between diel and dark treatments. Circled point was identified as an outlier (SYSTAT 12). Clone line variability over time should be noted, specifically looking at >80 hours (b) and that clones with the lowest survival at 160+ hours (b) are typically associated with differences in diel and dark tolerances (c) over the course of the experiments.

Based on the Bayesian GLMM analysis, expected overall Day 7 survival was 0.95 (0.90, 0.98, credible intervals, Table 1) in the diel treatment vs. 0.71 (0.52, 0.86) in the dark treatment. Survival probabilities in the diel-only treatment were in general higher and less variable (0.91–0.96; Table 1) than in the dark treatment (0.35–0.89; Table 1). Lower survivorship in the dark treatment compared to the diel treatment was most pronounced for clones B28, A60, A49, B45, A31, and B44 (Fig 3A and 3B).

**Table 1:** The Survival Probabilities of the Diel-Only Treatment.

	**Survival Probability**	
**Clone**	**Light**	**Dark**
B34	0.96 (0.88, 0.99)	0.89 (0.74, 0.98)
A52	0.95 (0.88, 0.99)	0.85 (0.69, 0.96)
B42	0.91 (0.77, 0.97)	0.73 (0.54, 0.88)
A69	0.95 (0.87, 0.99)	0.82 (0.65, 0.94)
B28	0.95 (0.87, 0.99)	0.70 (0.51, 0.86)
A60	0.96 (0.90, 1.00)	0.75 (0.56, 0.90)
A49	0.96 (0.89, 1.00)	0.63 (0.43, 0.81)
B45	0.94 (0.85, 0.99)	0.58 (0.38, 0.77)
A31	0.94 (0.85, 0.99)	0.58 (0.38, 0.76)
B44	0.91 (0.78, 0.98)	0.35 (0.15, 0.56)

Mean (95% credible interval) of estimated posterior distributions for clone-specific survival by treatment.

Fecundity (cumulative number of neonates produced; Fig 3C) was measurably different between diel and dark treatments for clones A 69 and A 34 (Table 2), both of which exhibited high survivorship in the dark treatment (Fig 3B). Clonal survivorship in the dark treatment exhibited a significant, positive relationship with the proportional difference between fecundity of clones in the L:D and dark treatments (Fig 3D; R^2^ = 0.451, P = 0.033). Clones that produced fewer neonates in dark compared to L:D treatment tended to have higher survivorship in the dark treatment. This relationship was even stronger when the identified outlier (Clone A52) was removed from the regression (R^2^ = 0.671, P = 0.007).

**Table 2:** Comparison of Mean Cumulative Fecundity Between the Dark and Diel Treatments (7-day).

	**Dark treatment**	**L:D treatment**			
**Clone**	**Mean (SD) fecundity**	**Mean (SD) fecundity**	**df**	**t**	**p**
A31	10.7 (4.9)	11.5 (4.9)	21.5	-0.4	0.683
A49	15.8 (4.1)	18.6 (5.8)	30.9	-1.6	0.116
A52	12.5 (5.9)	13.8 (5.4)	35.7	-0.7	0.463
A60	10.0 (4.5)	12.3 (5.2)	33.7	-1.4	0.171
**A69**	**15.2 (6.0)**	**23.8 (5.0)**	**31.4**	**-4.6**	**0.000**
B28	12.7 (9.1)	19.4 (12.8)	32.2	-1.8	0.082
**B34**	**16.9 (8.6)**	**23.1 (8.0)**	**35.8**	**-2.297**	**0.028**
B42	11.3 (3.9)	14.7 (8.6)	21.1	-1.440	0.165
B44	14.8 (11.2)	13.5 (9.7)	5.6	0.235	0.822
B45	17.3 (6.5)	17 (18.2)	21.7	-0.364	0.719

Results of two-sample T-tests comparing mean cumulative fecundity between the dark and diel treatments for individuals surviving the entire 7-day experiment. Clones that exhibited a statistically significant difference between the dark and diel treatments are in bold font.

### UV tolerance experiment

**Fig 4:**
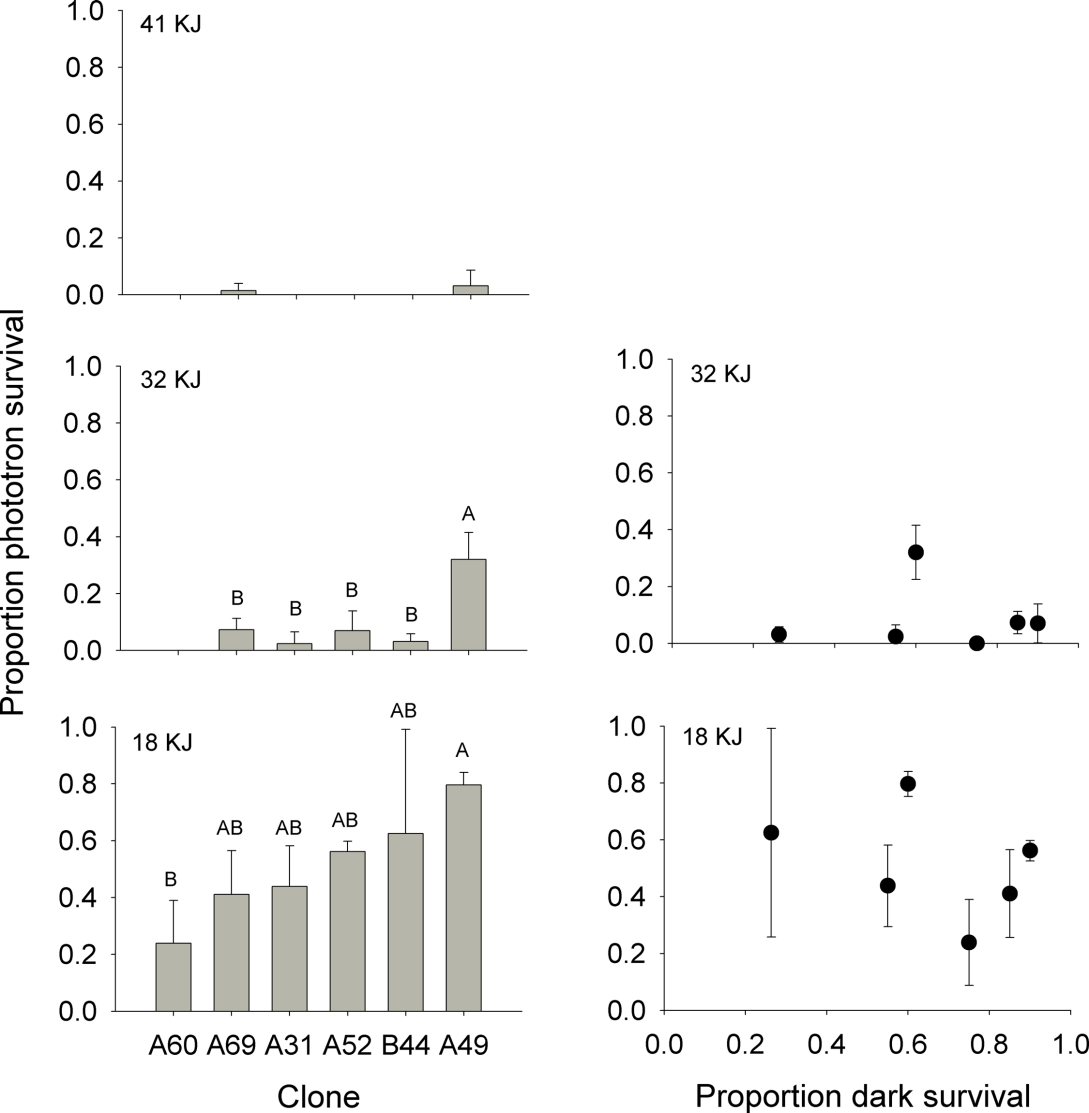
UV tolerance fitness. Left panels: Proportion survival of six *D*. *parvula* clones at Day 5 after phototron exposure to 41, 32, and 18 KJ. Right panel: Relationship between proportion survival after phototron exposure and proportion survival in the dark treatment for six clones. Graph for 41 KJ omitted due to uniform low survival across all clones. Each bar and point represents the mean of three phototron experiments per given clone. Error bars represent ± 1 STD. Letters represent statistically significant differences between clones. Variable UV tolerance is often observed across species of *Daphnia* but has not been emphasized within clone lines to this degree.

While we observed a predictable increase in survival with decreased UVR exposure, within those data were significant differences among clone lines. In the 41 KJ UV-B treatment, survival at 5 d post-exposure was universally low among clones with 4 of the 6 clones exhibiting 100% mortality (Fig 4). In the 32 KJ UV-B treatment, there was a significant difference in 5 d survival among clones (ANOVA; *F*(5,12) = 14.062, *P* < 0.001), with clone A49 exhibiting higher survivorship than any other clone (Tukey HSD, *P* < 0.05) (Fig 4). In the 18 KJ UV-B treatment, there was a significant difference in 5 d survival among clones (ANOVA; *F*(5,12) = 3.272, *P* = 0.043) with clone A49 exhibiting higher survival than clone A60 (Tukey, HSD *P* < 0.05), but all other clones exhibiting intermediate survival between clones A49 and A60 (Tukey HSD, *P* > 0.05; Fig 4).

Linear regression analysis revealed no significant relationship between 5-day post-exposure survival and proportion dark survival of the six clones for either the 32 KJ UV-B treatment (R^2^ = 0.000, *P* = 0.994) or the 18 KJ UV-B treatment (R^2^ = 0.144, *P* = 0.459). Regression analysis was not done for the 41 KJ UV-B treatment due to uniformly low 5-day post-exposure survival.

## Discussion

We have investigated the variable responses of clone lines under experimental conditions in a laboratory setting. This focus has increased our understanding of the potential for variable results in large population studies that are otherwise controlled (e.g. temperature, pH, and food availability). Further, these findings presented herein should be considered to be a foundation for a variety of future studies, both in the laboratory and in the field. Additional studies should be conducted focusing on the behaviors of *Daphnia* under dark conditions (feeding rates, mobility, etc.) and on the proteomics of the clone lines (e.g. are some clone lines more adept at up-regulating proteins needed to divert energy to fecundity?).

While a host of information regarding the negative effects of UVR on organisms, and more recently a growing body of evidence demonstrating positive effects of UVR for some, are readily available, evidence of negative effects of constant darkness on organisms is lacking outside of the realm of circadian rhythms and sleep patterns (e.g. temperature limit variability with altered circadian rhythms in *Drosophila* [17] and the impacts of light pollution on the circadian rhythms of humans and natural populations [18]). The findings presented herein extend effects of low- or no-light conditions beyond circadian rhythms, and demonstrate that both light extremes–too much or too little–have negative consequences. These results suggest that there is some optimum light condition that is required for *D*. *parvula*¸ and that these optima may vary among clones.

Certain chemicals, including cadmium and copper, have been reported by Barata et al. [19] and Agra et al. [20], respectively, to have a range of effects among clones, suggesting that there may be strong clonal selection under given conditions and may be indicative of considerable genetic variability. Agra [20] reports up to a 48-fold increase in tolerance for copper resistant clones as compared to sensitive clone lines, including higher growth rates and faster reproduction in the tolerant lines. Clonal variation has been identified in multiple studies of behavioral avoidance (e.g. [21–22]) and pigmentation (e.g. [23]). Spitze [24] specifically notes clonal responses to predation in *D*. *pulex*, and calls for studies that focus on genetic variability in prey vulnerability. Previous experiments have also noted variability of invertebrate fitness in diel experiments and have suggested that food availability was a key limitation (e.g. [25]).

Numerous studies over the past 20 years have demonstrated the positive effects of UV-A and visible light in the DNA repair processes of organisms for which photolyase enzymes are present (e.g. [26–28]), but the number of organisms with such enzymes is extremely limited. The more recent suggestions in the literature that the indirect effects of UVR can also be impactful for organisms allows a much broader net to be cast when considering the implications of changing UVR and climate variability. For example, several groups have more recently investigated the potential positive impact of UVR on reducing parasitic populations in aquatic systems, concomitantly improving the health of host populations (e.g. [10, 29]). Lafferty and Holt [30] describe how decreases in aquatic parasites, not only their numbers, but also their infectivity rates, should consistently result in decreases in infected hosts in a system. Their modeling has shed light on the potential of host-specific versus non-specific infections in stressed host populations, where stress included thermal, food, and population density factors. While these factors been investigated and known for decades in studies of human waterborne pathogens (e.g. [31]), it is only more recently been applied to non-human parasite investigations. As Overholt et al. [10] suggest, the relative tolerance of a host (e.g. *Daphnia*) and its parasite (fungal or other) to UVR may be one of the most important questions when investigating the potential positive effects of UVR.

The presence of UVR, at least in low to moderate levels, is potentially critical to the fitness of natural populations. In this study, clones that reduced fecundity in total darkness had increased survivorship (Fig 3). While the mechanism for this is unknown, and should be considered in future studies, the presence of diel cycles is likely driving the energy balance (acquisition and use) in species. Overholt et al. [32] have demonstrated variability in the short-term behavioral responses of copepod species to UVR, and suggest their attraction to UVR may be resource and/or competition avoidance driven. While other studies have suggested similar drivers of positive phototaxis, the presence or absence of light has also been shown to induce plastic variability in aquatic species. Effertz and von Elert [33] have demonstrated a significant effect of light intensity on life-history changes in *Daphnia magna*. They have shown that in the presence of fish kairomones and light there is a reduction in the size at first reproduction in *D*. *magna* compared to the presence of fish kairomones in the absence of light. The latter showed no significant effect on size at first reproduction, suggesting the plasticity of this life-history change is driven, at least in part, by the presence or absence of light. Further, with the advancement of genomic analyses, studies such as Jansen et al. [34] are demonstrating that rapid rates of evolution and altered gene expression in some species might well pave the way for even higher variability in life-history traits and may be driven by the presence or absence of high intensity light in resurrected and present day populations (e.g. *Daphnia magna*).

There are three specific broader applications that should be considered from our findings. The first is that greater attention to both the positive effects of UVR/light and clonal variability in laboratory controlled studies, mesocosm research, and full-scale system investigations will be vital to our understanding of ecosystem dynamics. Secondly, while a multitude of previous studies have demonstrated the avoidance of high light conditions in many organisms, including some of our own work, the concept that the avoidance of dark might well be an advantage to some species is worth further consideration. The recent work by Overholt et al. [10], Paull and Johnson [35], and others focusing on the positive impacts of abiotic stresses (including UVR) on host species in parasite and fungal-rich ecosystems is of particular interest. The extension of this parasite-host interaction work to terrestrial ecosystems should be considered. And lastly, the newer application of genomics and proteomics to improve our understanding of the evolutionary changes, and potentially highly plastic phenotypic expression of life-history changes and organism tolerance to abiotic and biotic stressors must be expanded.
